# Characterizing academic performance in pediatric acute lymphoblastic leukemia with population‐based achievement tests

**DOI:** 10.1002/cnr2.1560

**Published:** 2021-09-30

**Authors:** Hend M. Al‐Kaylani, Erin E. Reasoner, Bradley T. Loeffler, Sarah L. Mott, Susan Madasu, Audrey Liu, Kathleen Langbehn, Amy L. Conrad, David Dickens, Amanda Grafft, Lyndsay Harshman, Arunkumar J. Modi, Ellen van der Plas

**Affiliations:** ^1^ Department of Psychiatry University of Iowa Hospital and Clinics Iowa City Iowa USA; ^2^ Holden Comprehensive Cancer Center University of Iowa Hospital and Clinics Iowa City Iowa USA; ^3^ Stead Family Department of Pediatrics University of Iowa Hospital and Clinics Iowa City Iowa USA; ^4^ Department of Health Education and Behavior University of Florida Gainesville Florida USA; ^5^ Department of Pediatrics University of Arkansas for Medical Sciences Little Rock Arkansas USA

**Keywords:** cancer, cognition, education, leukemia, oncology, survivors

## Abstract

**Background:**

Recent shifts from radiation to chemotherapy‐based treatment for acute lymphoblastic leukemia (ALL) have contributed to reduced long‐term morbidity. Despite this, ALL survivors remain at increased risk for long‐term cognitive impairments.

**Aim:**

To identify demographic and treatment factors associated with school performance in pediatric survivors of ALL.

**Methods:**

We collected standardized test scores for reading, math, and science obtained in a school setting from grades 3–11 in 63 ALL survivors (46.0% boys). Most participants were assessed across multiple grades (median number of grades *n* = 5, range 1–7), and 269 observations were considered in the analyses. Treatment exposures were extracted from medical records. Socio‐economic status was estimated using participation in free/reduced lunch programs at school. Mixed effects linear regression models were conducted to determine factors associated with school performance.

**Results:**

ALL survivors' scores were comparable to state norms on reading, math, and science performances. On multivariable analysis, participation in free/reduced lunch programs was significantly associated with lower reading scores (*β* = −12.52; 95% CI −22.26:−2.77, *p =* .01). Exposure to radiation during treatment was also associated with lower reading test scores (*β* = −30.81, 95% CI −52.00:−9.62, *p* = .01). No significant associations between demographics and treatment parameters were observed for math and science test scores.

**Conclusions:**

We utilized population‐based achievement tests conducted from grades 3–11 to characterize school performance in ALL survivors. Our results imply that survivors with low socio‐economic status and those exposed to radiation during treatment could benefit from early monitoring and intervention to maximize academic success.

**Abbreviations**
ADHD: attention deficit/hyperactivity disorder
ALL: acute lymphoblastic leukemia
CNS: central nervous system
IDE: The Iowa Department of Education
IT: intrathecal
MTX: methotrexate
PO: oral
SES: socio‐economic status

## 1 INTRODUCTION

Acute lymphoblastic leukemia (ALL) accounts for approximately 25% of childhood cancers and is most frequently diagnosed between 2 and 5 years of age.^1^ While uniformly fatal prior to the 1960s, close to 95% of patients are currently expected to survive. Cranial radiation‐based protocols have largely been replaced with systemic and intrathecal (IT) therapies for central nervous system (CNS) prophylaxis.^2^ The shift to intrathecal therapies has contributed to improved neurocognitive outcomes; yet, ALL survivors treated with non‐radiative chemotherapy‐based protocols remain at risk for cognitive difficulties and academic underachievement^3^ that impact long‐term quality of life.^4^, ^5^

Various studies report evidence of low‐average academic performance and reduced academic attainment among ALL survivors.^6^, ^7^, ^8^, ^9^ Current evidence is primarily predicated using psychological tests to assess survivors' performance, including the Wechsler Individual Achievement Test and the Wide Range Achievement Test.^6^, ^8^, ^10^, ^11^ While these tests are psychometrically robust, there are notable limitations regarding ecological validity,^12^, ^13^ as these measures are typically administered in a quiet testing space with a dedicated examiner. By contrast, most school activities occur while experiencing significant environmental distractions. Little work has been done to evaluate academic performance of survivors in a real‐word setting.^12^

Harshman and colleagues^14^ characterized academic achievement in ALL survivors using data obtained through statewide testing in schools. They demonstrated that ALL survivors who were diagnosed between 1993 and 2008 exhibited lower scores in mathematics. However, the authors had limited access to patient information, and no inferences could be made about potential risk factors of academic underachievement.

Risk factors of poor school performance may derive from a wide contextual spectrum, including treatment exposures, physiological factors, and social domains.^15^ Recent work has highlighted the importance of socio‐economic status (SES) as a key consideration in academic performance. In the United States, SES can be approximated by utilization of public health insurance,^16^ and recent studies have shown that ALL survivors on public health insurance had more academic difficulties than survivors on private insurance.^10^, ^15^, ^16^ Identifying determinants of school performance in ALL survivors will broaden the scope and depth of our understanding of academic achievement following ALL.

The objective of the present study was to characterize academic outcomes of patients with ALL treated between 2000 and 2019 using reading, math, and science scores obtained in a school setting. Associations between academic outcomes and treatment exposures, risk stratifications, age at diagnosis, and socio‐economic status were explored. We hypothesized that ALL survivors would exhibit reduced performance in math relative to state norms, and that younger age at diagnosis, high‐risk treatment, and lower socio‐economic status would be associated with academic performance.

## 2 METHODS

### 2.1 Study population

**FIGURE 1:**
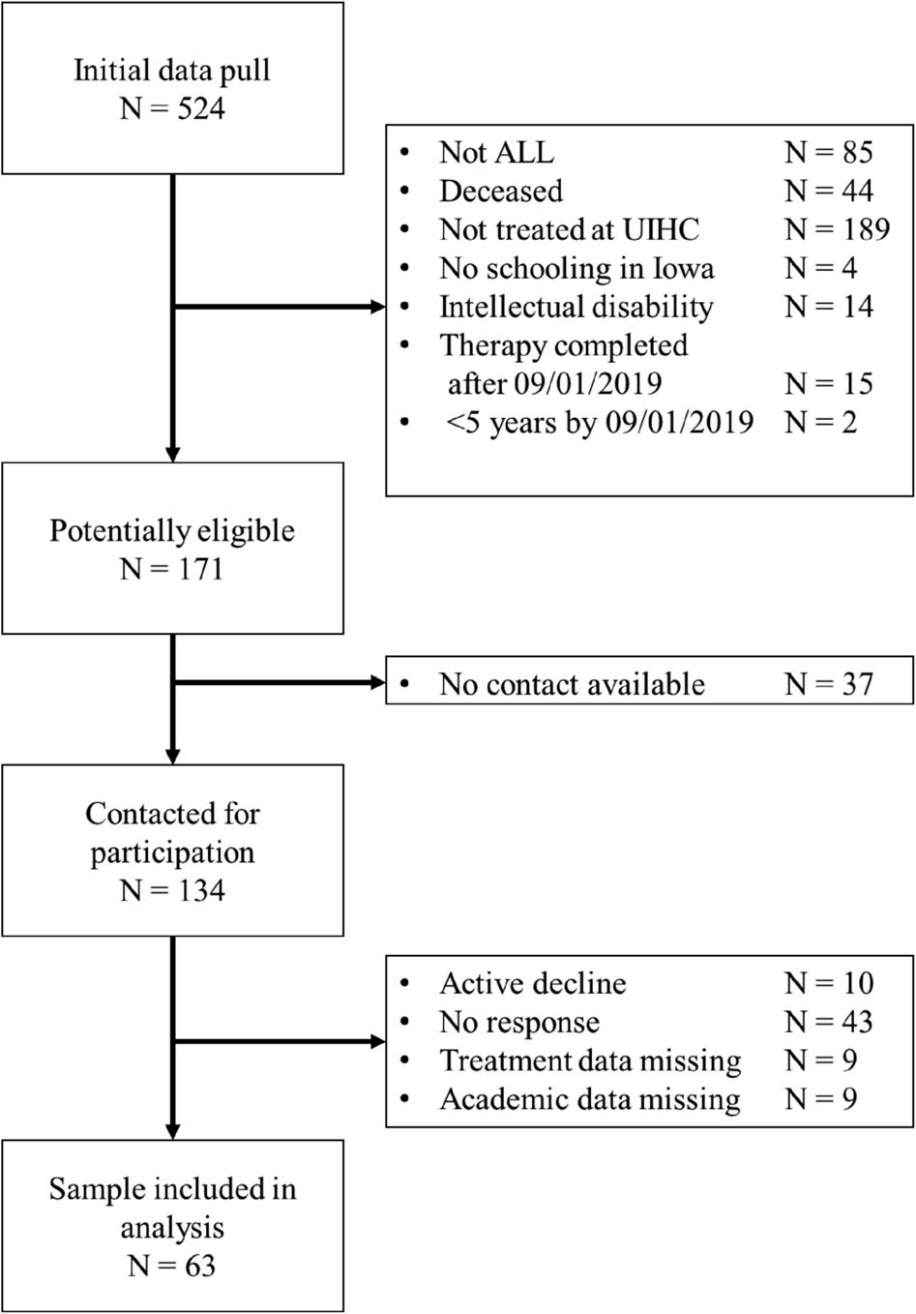
Consort diagram. Of potentially eligible participants, 91 (53.2%) responded. The final sample included 63 individuals

Potentially eligible participants treated at the University of Iowa were identified through medical records. Inclusion criteria included: (1) diagnosis of ALL; (2) treatment received between January 1, 2000 and September 1, 2019 at our institute; (3) between 0 to 18 years of age at diagnosis; (4) completed at least one Iowa Assessment at school; (5) did not have any conditions associated with significant intellectual disability (e.g., trisomy 21); and (6) were ≥5 years of age as of September 1, 2019. Participants with both academic and treatment data were included in the analysis (Figure 1).

### 2.2 Outcome variables

The Iowa Assessments are validated achievement tests designed to identify students' strengths and weaknesses, monitor growth, and predict future performance.^17^ Foundational skills encompassing reading, math, and science are measured annually in public and private schools in the state of Iowa. The Iowa Assessments were derived from extensive, iterative testing in representative national and state samples. High concurrent‐ and predictive validity has been established for the Iowa Assessments.^18^ Notably, Iowa Assessments scores were strongly associated with ACT scores.^17^, ^18^ Additionally, excellent reliability coefficients across grades have been reported, with composite coefficients ranging from 0.927 to 0.983.^18^

Statewide data are maintained by the Iowa Department of Education (IDE), and encompass test scores, attendance, and resources utilized by individual students (i.e., Section 504 plan, free/reduced lunch). The primary outcomes of interest for the present study encompassed test scores for reading, math, and science that were obtained in academic years 2011–2012 through 2017–2018.

Three levels of achievement are defined, including: not proficient (scores that fall in the lowest quartile of the distribution), proficient (score that fall within quartiles 2 and 3 of the distribution), and advanced (scores that fall in the highest quartile of the distribution).^19^ The Iowa Department of Education (IDE) provided the overall state means and standard deviations for reading, math, and science for comparison purposes. Per our agreement with the IDE, state means will not be specified on plots.

### 2.3 Predictors

#### 2.3.1 Demographics

Demographic variables in this analysis included sex and SES. The IDE provided information about participation in free/reduced‐price lunch at school, which was used as a proxy for SES. Free/reduced‐price lunch programs were launched to ensure poor students in the US are adequately nourished, and the program is instrumental in determining allocation of funds to schools that educate economically disadvantaged youth.^20^ Education researchers routinely use free/reduced‐price lunch enrollment as a proxy for economic disadvantage.^21^

#### 2.3.2 Treatment parameters

Detailed treatment exposures were obtained through medical records and included: (1) radiation exposure (yes/no); (2) bone marrow transplant (yes/no); (3) cytarabine; (4) asparaginase; (5) steroids; (6) intravenous methotrexate (IV MTX); (7) intrathecal (IT) MTX; (8) vincristine; and (9) anthracyclines.

Cumulative exposure to asparaginase was derived from asparaginase (IU/m^2^), L‐asparaginase (IU/m^2^), PEG‐asparaginase (IU/m^2^) and Erwinia (IU/m^2^). PEG‐asparaginase dose equivalencies were calculated for Erwinia, as the latter is immunologically distinct from other *Escherichia coli*‐derived Asparaginases and requires differential dosing.^22^ Based on previous work,^23^ cumulative dose of Erwinia was divided by 60 to estimate the PEG‐asparaginase equivalent.

Cumulative dose of corticosteroids was calculated from oral (PO) dexamethasone (mg/m^2^) and prednisone PO (mg/m^2^). Dexamethasone equivalency for prednisone was calculated based on COG long‐term guidelines (version 5.0), where prednisone dose was divided by 6.25.

The cumulative anthracycline dose was derived from doxorubicin IV (mg/m^2^) and daunorubicin IV (mg/m^2^). Following COG long‐term guidelines (version 5.0), the cumulative dose for daunorubicin was multiplied by 0.5 to establish a doxorubicin dose‐equivalency.

Other relevant treatment‐related parameters that were extracted from medical records included age at diagnosis, age at the end of treatment, and risk stratification (standard vs. high).

### 2.4 Statistical analysis

Linear mixed effects models were conducted to evaluate associations between demographic variables, treatment characteristics, and school performance. Models were adjusted for the baseline trend in test scores across grade level. Overall non‐proficiency rates were estimated using logistic mixed effects models. Random effects were included to account for the correlated nature of repeat assessments within participants over time in all models. All available observations were included in each model. Only variables that were significant on univariate analysis were included in the multivariable model. All statistical testing was two‐sided and assessed for significance at the 5% level using R v4.0.2 (https://www.R-project.org/).

## 3 RESULTS

### 3.1 Sample

The final sample included 63 individuals (46.0% boys) who provided 269 observations (median number of grades *n* = 5, range 1–7). There was an equal distribution of boys and girls (*χ* ^2^_(1)_ = 2.2, *p* = .14). Of 63 individuals, 24 (38.1%) had neurodevelopmental conditions noted in their medical charts, including attention deficit/hyperactivity disorder (ADHD; *n* = 6), learning difficulty (*n* = 6), or mild neurocognitive impairment (*n* = 3). The remainder (*n* = 9) presented with a combination of these conditions (Figure S1).

Table 1 describes demographic characteristics and treatment information. Specific treatment protocols are shown in Table S1. Cumulative treatment exposures are shown in Table S2. Participation in school programs (e.g., individual education plan) across grades are presented in Table 2.

**TABLE 1:** Demographics and basic treatment characteristics

Data type	Variable	Level	Statistics		
Demographics	Sex	Boys: *N* (%)	29 (46.0)		
		Girls: *N* (%)	34 (54.0)		
	Race/Ethnicity	White: *N* (%)	51 (81.0)		
		Black: *N* (%)	3 (4.8)		
		Hispanic: *N* (%)	7 (11.1)		
		Other: *N* (%)	2 (3.1)		
Academic data	Observations across grade and outcome measure	Grade	Reading	Math	Science
		3	37	38	35
		4	37	38	35
		5	31	32	31
		6	34	34	33
		7	32	32	32
		8	32	32	32
		9	23	23	23
		10	20	20	20
		11	20	19	20
ALL treatment	ALL diagnosis	B‐cell: *N* (%)	55 (87.3)		
		T‐cell: *N* (%)	5 (7.9)		
		Bi‐phenotypic: *N* (%)	3 (4.8)		
	Age at ALL diagnosis	Mean (SD)	5.1 (3.4)		
	Risk stratification	Standard risk: *N* (%)	36 (57.1)		
		High risk: *N* (%)	27 (42.9)		
	Radiation	No: *N* (%)	51 (81.0)		
		Yes: *N* (%)	12 (19.0)		
	Bone marrow transplant	Yes: *N* (%)	5 (7.90)		
		No: *N* (%)	58 (92.1)		
	Undergoing treatment^a^	Yes: *N* (%)	15 (23.8)		
		No: *N* (%)	48 (76.2)		

^a^ Undergoing treatment for at least one grade.

**TABLE 2:** School program participation and proficiency level of scores across grades

Variable	Stat	Level	3 (*N* = 38)	4 (*N* = 38)	5 (*N* = 32)	6 (*N* = 34)	7 (*N* = 32)	8 (*N* = 32)	9 (*N* = 23)	10 (*N* = 20)	11 (*N* = 20)
IEP?^a^	*N* (%)	Yes	7 (18.4)	8 (21.1)	7 (21.9)	9 (26.5)	5 (15.6)	6 (18.8)	2 (8.7)	3 (15.0)	3 (15.0)
	*N* (%)	No	31 (81.6)	30 (78.9)	25 (78.1)	25 (73.5)	27 (84.4)	26 (81.2)	21 (91.3)	17 (85.0)	17 (85.0)
Free/Reduced Lunch?	*N* (%)	Yes	12 (31.6)	14 (36.8)	14 (43.8)	16 (47.1)	15 (46.9)	14 (43.8)	7 (30.4)	7 (35.0)	5 (25.0)
	*N* (%)	No	26 (68.4)	24 (63.2)	18 (56.2)	18 (52.9)	17 (53.1)	18 (56.2)	16 (69.6)	13 (65.0)	15 (75.0)
Gifted/talented?	*N* (%)	Yes	3 (7.9)	6 (15.8)	6 (18.8)	3 (8.8)	4 (12.5)	3 (9.4)	3 (13.0)	1 (5.0)	2 (10.0)
	*N* (%)	No	35 (92.1)	32 (84.2)	26 (81.2)	31 (91.2)	28 (87.5)	29 (90.6)	20 (87.0)	19 (95.0)	18 (90.0)
504 plan?^b^	*N* (%)	Yes	2 (5.3)	3 (7.9)	2 (6.2)	1 (2.9)	2 (6.2)	4 (12.5)	6 (26.1)	5 (25.0)	2 (10.0)
	*N* (%)	No	36 (94.7)	35 (92.1)	30 (93.8)	33 (97.1)	30 (93.8)	28 (87.5)	17 (73.9)	15 (75.0)	18 (90.0)
Accumulated present days	*N*		38	37	32	34	32	32	23	20	20
	Mean		153.1	167.1	168.2	168.2	167.8	158.7	168.1	164.5	152.1
	SD		32.8	11.2	8.0	6.9	7.6	24.7	10.9	14.7	46.9

^a^ Individualized education plan.

^b^ Plan developed to provide appropriate accommodations for a child with disabilities attending elementary or secondary school.

### 3.2 Reading scores

**FIGURE 2:**
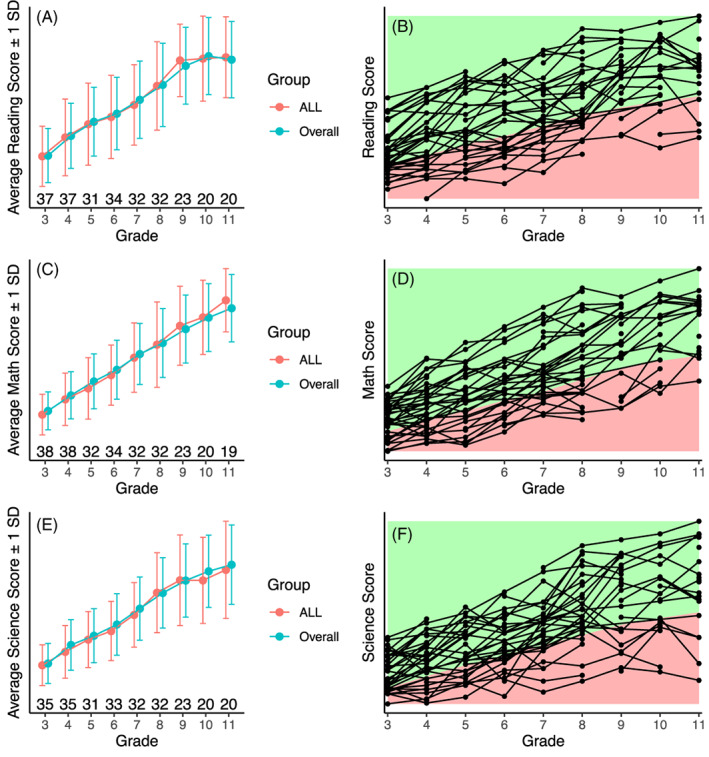
Academic achievement scores across grades 3 through 11. Panels A and B depict reading scores, panels C and D show math scores, and panels E and F show science scores. Left‐sided panels show average standard scores (y‐axis) across grades (x‐axis) for ALL survivors (pink) and across the state (blue). The number of observations for ALL survivors are included for each grade. Right‐sided panels show ALL performance across grades, where the green areas demarcate scores that are in the proficient range, whereas red‐shaded areas demarcate non‐proficient scores. As per our agreement with IDE, the y‐axis was left unmarked

Average reading scores of ALL survivors across grades 3–11 were similar to statewide norms (Figure 2A). Scores across grades within individuals are shown in Figure 2B. The estimated rate of non‐proficient reading scores of the sample was 27.3% (95% confidence interval [CI] 17.7:39.4), which was within the expected range of non‐proficiency. Proficiency rates for reading across grades are shown in Table 3.

**TABLE 3:** Proficiency rates of reading, math, and science across grades

Domain	Outcome	Stat	Level	3 (*N* = 38)	4 (*N* = 38)	5 (*N* = 32)	6 (*N* = 34)	7 (*N* = 32)	8 (*N* = 32)	9 (*N* = 23)	10 (*N* = 20)	11 (*N* = 20)
Reading	Proficient?^a^	*N* (%)	No	10 (27.0)	13 (35.1)	10 (32.3)	15 (44.1)	11 (34.4)	13 (40.6)	3 (13.0)	4 (20.0)	4 (20.0)
		*N* (%)	Yes	27 (73.0)	24 (64.9)	21 (67.7)	19 (55.9)	21 (65.6)	19 (59.4)	20 (87.0)	16 (80.0)	16 (80.0)
		*N* (%)	Missing	1	1	1	0	0	0	0	0	0
Math	Proficient?^a^	*N* (%)	No	12 (31.6)	13 (34.2)	10 (31.2)	11 (32.4)	9 (28.1)	11 (34.4)	5 (21.7)	4 (20.0)	2 (10.5)
		*N* (%)	Yes	26 (68.4)	25 (65.8)	22 (68.8)	23 (67.6)	23 (71.9)	21 (65.6)	18 (78.3)	16 (80.0)	17 (89.5)
		*N* (%)	Missing	0	0	0	0	0	0	0	0	1
Science	Proficient?^a^	*N* (%)	No	10 (28.6)	11 (31.4)	9 (29.0)	12 (36.4)	9 (28.1)	5 (15.6)	7 (30.4)	8 (40.0)	5 (25.0)
		*N* (%)	Yes	25 (71.4)	24 (68.6)	22 (71.0)	21 (63.6)	23 (71.9)	27 (84.4)	16 (69.6)	12 (60.0)	15 (75.0)
		*N* (%)	Missing	3	3	1	1	0	0	0	0	0

^a^ Cutoff scores vary by grade.^19^

Participation in free or reduced lunch programs was significantly associated with reduced reading scores (*β* = −12.52, 95% CI −22.26:‐2.77, *p* = .01). Treatment exposures also modulated reading scores. Having received radiation treatment was associated with reading performance (*β* = −30.81, 95% CI −52.00:−9.62, *p* = .01). While not statistically significant at *p* < .05, univariate models indicated that exposure to high‐risk treatment protocols tended to be associated with lower reading scores (*β* = −16.61, 95% CI −34.57:1.35, *p* = .07). Other relevant treatment variables, including age at diagnosis and cumulative exposures to chemotherapy agents, were not significantly associated with reading. Summary statistics for all predictors that were considered in the univariate analyses are shown in Table S3.

### 3.3 Math scores

As shown in Figure 2C, math performance of participants approximated state normative data. The sample's estimated rate of non‐proficiency in math was within the expected range: 27.4% (95% CI 19.3:37.4). Table 3 includes proficiency rates for math across grades. None of the predictor variables were significantly associated with math performance (Table S4).

### 3.4 Science scores

Figure 2E shows science scores of participants relative to state means, showing that ALL participants had similar scores as grade averages. The rate of non‐proficient science scores was 28.1% (95% CI 19.7:38.3). Science proficiency across grades is shown in Table 3.

Univariate analysis showed that the pattern of association between free/reduced lunch and science scores was similar to that of reading scores, but did not reach threshold of statistical significance at *p* < .05 (*β* = −7.79, 95% CI −16.78:1.19, *p* = .09).

Treatment variables were not significantly associated with science scores. Summary statistics for all predictors are shown in Table S5.

## 4 DISCUSSION

A key strength of the present study was the use of school data to characterize academic performance following childhood ALL. We gathered important indicators of SES and collected information about treatment exposures. Our findings suggest that academic performance is within normal limits among children and adolescents with a history of ALL. However, low SES and radiation treatment are risk factors of reduced academic achievement.

School meal programs were established to support students from low‐income families. According to the US Department of Education, children in households with incomes at or below 130% of the federal poverty level are eligible for free school meals. This measure is routinely used in the education literature as a proxy for economic disadvantage.^21^ Participants who received free or reduced lunch at school scored significantly lower on reading than those who did not participate in school meal programs. The magnitude of the score reduction was roughly equal to being approximately 1 grade behind peers, on average. Our results are in line with previous literature showing that ALL survivors who were covered through public insurance were at higher risk of exhibiting neurocognitive difficulties.^10^, ^16^ Comparable metrics are applied to determine eligibility for US public health insurance coverage. We opted to use free/reduced lunch because it was collected at the same time as our outcome measures. Further, individuals with private insurance may also be eligible for public insurance, complicating the interpretation as a proxy for SES. Few studies have systematically evaluated socio‐economic risk factors of academic difficulties in childhood ALL survivors, highlighting opportunities for further research.^15^

The Children's Oncology Group Long Term Follow‐Up identifies cranial radiation as a prominent risk factor of neurocognitive deficits following childhood cancer.^24^ In line with this, children and adolescents who had been exposed to radiation scored substantially lower on reading tests than those who were not exposed to radiation. The reduction in reading scores of radiated ALL survivors was equivalent to approximately two grades below current grade level. Since radiation exposure is known to affect the developing brain,^25^, ^26^ it is not surprising that exposure to radiation interferes with neurocognitive abilities and academic success.^27^ The magnitude of the academic deficit points to the critical importance of continued monitoring of radiated ALL survivors.

Exposure to high‐risk protocols followed a similar trend as radiation exposure, where participants scored lower than those who had received a standard‐risk protocol on reading. However, cumulative exposure to specific chemotherapy agents did not affect school performance in this sample. The evidence regarding the impact of treatment burden on neurocognitive outcomes is conflicting, with some studies showing significant associations between exposure to MTX or corticosteroids,^28^, ^29^ while other studies did not.^8^, ^30^ The lack of association between cumulative treatment exposures and school performance observed in the present study may be due to difficulties detecting the individual contributions of agents that were administered in a combination and via various routes.^8^, ^28^, ^31^

Younger age at diagnosis has commonly been found to be associated with poorer neurocognitive outcomes following childhood ALL.^9^, ^15^ For example, Jacola and colleagues reported that survivors diagnosed prior to age 10 had lower math scores than survivors who were diagnosed at an older age.^10^ Likewise, Harshman and colleagues showed that survivors diagnosed prior to the age of 5 years old had reduced academic achievement relative to those diagnosed after the age of 5.^14^ By contrast, we observed no significant association between academic outcomes and age at diagnosis. These results are in line with a recent study on neurocognitive impairment in a large cohort of ALL survivors.^32^ It is possible that the impact of age at diagnosis is modest among individuals treated with chemotherapy only,^33^ and 80% of the current sample was treated with chemotherapy alone. Further, individuals in the present sample were treated across nine different protocols, while Jacola and colleagues utilized a sample that was treated on the same protocol (AALL0232). A more homogeneous sample may be required to detect potentially modest effects of age at diagnosis.

While math skills are commonly reported to be affected in ALL survivors,^34^, ^35^ participants in the present sample scored within expectation on mathematics assessments. Using a similar approach, Harshman and colleagues^14^ reported that survivors who were treated between 1993 and 2008 scored significantly lower than the 50th percentile of state norms in 8th grade and 11th grade. The authors did not have access to information about treatment exposures in this cohort. However, given the time epoch at which individuals were treated, it seems reasonable to expect that a substantial proportion were exposed to radiation.^2^, ^36^, ^37^ The current cohort was treated more recently, and the great majority received chemotherapy only. The different composition of the cohorts regarding radiation exposures may have contributed to the differences in math performance across the two studies. As with our study, survivors treated with chemotherapy only performed within normal limits on a validated neuropsychological measure of math achievement.^10^ Replacing cranial radiation with chemotherapy‐only regimens has clearly been beneficial in reducing toxicity,^38^ underscoring the importance of continued research on reducing the long‐term impact of cancer treatment.

The results of this study should be interpreted within the context of its limitations. First, the final sample included 37% of the potentially eligible sample. The relatively limited inclusion rate was in large part due to the rate of non‐responders who were approached via mail (53%). Lower response rates raise legitimate concerns about generalizability of the findings. Notably, key characteristics of the sample were representative of the population from which it was drawn. For instance, the distribution of race/ethnicity of the sample is representative of the Midwestern population where the study was conducted.^39^ Additionally, treatment characteristics are representative of pediatric ALL patients treated in the past two decades.^4^ Nonetheless, our sample size was limited, potentially affecting our ability to detect associations between risk factors and school performance. Relatedly, we could not obtain complete longitudinal school data on the entire sample, in part because some participants had yet to complete school. It will be important to replicate these findings in a larger and ethnically diverse sample of patients with ALL, and to expand this approach to other patient populations.

Second, while we collected detailed treatment information in our sample, we did not have sufficient data to explore associations between neuropsychological test performance during treatment and school performance. One study reported that visual‐motor abilities during treatment for ALL predicted academic outcomes in early survivorship as measured with the Woodcock‐Johnson Tests of Achievement.^11^ It will be important to consider neurocognitive function during treatment in future studies on academic performance among ALL survivors. This can enable the development of effective strategies of screening and monitoring to maximize school success.

Third, a small subset of participants were still undergoing treatment while they were attending school and completed the achievement tests. On average, these individuals missed 16 more school days relative to individuals who had already completed their treatment (149 days attended vs. 165 days attended in individuals undergoing treatment and survivors, respectively). The discrepancy in number of missed school days was relatively minimal and did not predict scores in univariate models. Overall, there was no statistically significant difference in school performance between individuals who were undergoing treatment and those who had already completed treatment.

Establishing collaborative partnerships with educational agencies provides invaluable perspectives on academic outcomes following pediatric cancer. Through the use of academic achievement data obtained in a school setting, we obtained a representative sample of individuals affected by ALL. Individuals from low SES backgrounds, individuals exposed to radiation, and those who required high‐risk protocols were at risk of underachievement in school. As a group, survivors performed similar to peers, which is a promising trend in view of efforts to minimize toxicity of treatment. However, there is evidence to suggest that survivors are less likely to graduate high school^9^ or to achieve an undergraduate degree.^7^ The negative impact of mild‐to‐moderate neurocognitive difficulties may amplify with age, as responsibilities and demands on executive functions increase.^3^ School performance is a strong predictor of quality of life among ALL survivors,^4^ underscoring the importance of continued research on developing individual plans that accommodate survivors' educational goals.^12^

## CONFLICT OF INTEREST

The authors have no conflicts of interest to disclose.

## AUTHOR CONTRIBUTIONS

All authors had full access to the data in the study and take responsibility for the integrity of the data and the accuracy of the data analysis. *Conceptualization*, E.v.d.P., L.H., and A.J.M.; *Methodology*, E.v.d.P., L.H., and A.J.M; *Investigation*, H.M.A., E.E.R., S.M., A.L., K.L., A.G., and A.J.M.; *Formal Analysis*, B.T.L. and S.L.M.; *Writing ‐ Original Draft*, H.M.A., E.E.R., and E.v.d.P.; *Writing ‐ Review & Editing*, H.M.A., E.E.R., A.C., D.D., A.G., L.H., A.J.M., and E.v.d.P.; *Visualization*, B.T.L. and S.L.M.; *Supervision*, E.v.d.P.; *Funding Acquisition*, E.v.d.P and A.J.M.

## ETHICAL STATEMENT

This study was approved by the Institutional Review Board for Human Subjects Research at the University of Iowa, which conforms to the standards of the Declaration of Helsinki. Consent, and/or assent, was obtained during appointments at the Stead Family Children's Hospital Cancer Survivorship Clinic or through mail‐in consent. Individuals ages 18 and older provided their own consent, while parents/guardians provided consent for individuals younger than 18. Dual consent from parents/guardians and participants was obtained for participants between 13 and 17 years old. Participants between the ages of 7 and 12 provided assent in addition to parental/guardian consent.

## Supporting information


**Appendix** S1: Supporting Information

## Data Availability

De‐identified data can be shared upon reasonable request.
