# Supplementary material for: Characterizing academic performance in pediatric acute lymphoblastic leukemia with population‐based achievement tests
Source: Cancer Rep (Hoboken). 2021 Sep 30;5(9):e1560. doi: 10.1002/cnr2.1560 (PMC9458490; doi:10.1002/cnr2.1560)
Supplement: Supplementary file 1 — Appendix S1: Supporting Information [file CNR2-5-e1560-s001.pdf]

Supporting Information for Review

SUPPLEMENTAL FIGURE 1 Euler diagram of comorbid neurodevelopmental conditions. Of 63 ALL survivors, 24 presented with one or more neurodevelopmental conditions, including Attention-Deficit/Hyperactivity Disorder (ADHD), Learning Difficulty, or Mild Neurocognitive Disorder. Ellipses representing diagnoses are proportional to the number of people who presented with the condition. Co-occurring conditions are represented by overlapping ellipses.

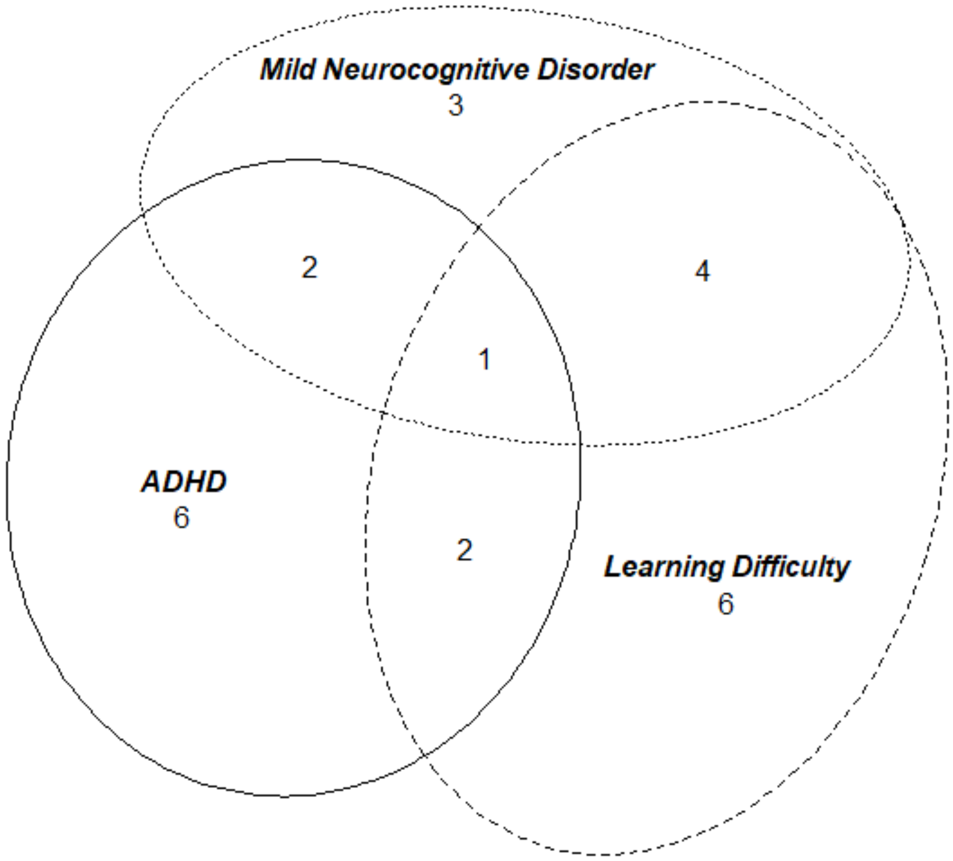

SUPPLEMENTAL TABLE 1 Treatment protocols included in the sample.

Protocol ID	N (%)
AALL0031	1 (1.6)
AALL0232	10 (15.9)
AALL0331	17 (27.0)
AALL0932	8 (12.7)
AALL1131	6 (9.5)
AALL1231	1 (1.6)
CCG1953	1 (1.6)
CCG1961	7 (11.1)
CCG1991	12 (19.0)

SUPPLEMENTAL TABLE 2 Treatment exposures.

Type of exposure	Agent Administered		Range	Dose
	**Yes: N (%)**	**No: N (%)**	**Min; Max**	**Mean (SD)**
Radiation to brain/spine (Gy)	12 (19.0)	51 (81.0)	12.0; 53.4	19.8 (11.0)
Cytarabine IV/SQ (mg/m^2)	61 (96.8)	2 (3.2)	600.0; 3600.0	2439.3 (5671.1)
Cytarabine IT (mg)	62 (98.4)	1 (1.6)	30.0:502.0	73.5 (60.4)
Asparaginase (IU/m^2)	63 (100.0)	0 (0)	2500.0; 90000.0	15,800.0 (18,988.2)
Steroids (mg/m^2)	62 (98.4)	1 (1.6)	210.0; 2113.6	1128.4 (349.1)
IV MTX (mg/m^2)	43 (68.3)	20 (31.7)	540.0; 45000.0	8029.5 (10208.9)
IT MTX (mg)	62 (98.4)	1 (1.6)	37.5; 432.0	241.5 (64.5)
PO MTX (mg/m^2)	59 (93.7)	4 (6.3)	220.0; 3080.0	1944.3 (636.5)
IV Vincristine (mg/m^2)	63 (100.0)	0 (0)	2.8; 99.0	59.1 (18.3)
Anthracyclines (mg/m^2)	63 (100.0)	0 (0)	50.0; 200.0	103.1 (39.6)

SUPPLEMENTAL TABLE 3 Univariate analyses for reading scores.

**Covariate**[a]	**Level**	**Beta**	**95% CI**		***P***
Free/Reduced Lunch?	Yes	-13.42	-23.35	-3.50	0.01
	No	Ref			
Sex	Girls	9.19	-8.93	27.31	0.31
	Boys	Ref			
Risk Stratification	High risk	-16.61	-34.57	1.35	0.07
	Standard risk	Ref			
Radiation?	Yes	-26.98	-49.50	-4.47	0.02
	No	Ref			
BMT?	Yes	-25.66	-57.85	6.53	0.12
	No	Ref			
Undergoing Treatment?	Yes	7.14	-3.50	17.78	0.19
	No	Ref			
Accumulated Present Days	Units=1 day	-0.03	-0.16	0.10	0.64
Cytarabine	Units=100 mg/m^2	-0.03	-0.19	0.12	0.70
Asparaginase	Units=100[b]	-0.03	-0.08	0.01	0.15
Steroids	Units=10[c]	-0.03	-0.17	0.10	0.61
Anthracyclines	Units=10[d]	-0.44	-1.76	0.88	0.51
Cumulative Cytarabine IT	Units=10 mg	-0.30	-1.52	0.93	0.63
Cumulative Methotrexate IV	Units=100 mg/m^2	0.01	-0.06	0.09	0.76
Cumulative Methotrexate IT	Units=10 mg	-0.01	-0.55	0.53	0.98
Cumulative Vincristine IV	Units=10 mg/m^2	0.62	-1.89	3.13	0.63
Age at treatment initiation	Units=1 year	-0.24	-3.03	2.55	0.86
Age at end of treatment	Units=1 year	-0.25	-3.00	2.51	0.86

[a]Adjusted for grade;

[b]100 IU/m^2 for Asparaginase IM, L-Asparaginase IM, PEG-Asparaginase; 6000 IU/m2 for Erwinia;

[c]10 mg/m^2 Dexamethasone PO; 62.5 mg/m2 Prednisone PO; [d]10 mg/m2 Doxorubicin IV; 20 mg/m^2 Daunorubicin IV.

SUPPLEMENTAL TABLE 4 Univariate analyses for math scores.

Covariate[a]	Level	Beta	95% CI		*P*
Free/Reduced Lunch?	Yes	-1.58	-8.88	5.72	0.67
	No	Ref			
Sex	Girls	-0.49	-12.68	11.70	0.94
	Boys	Ref			
Risk Stratification	High risk	-4.99	-17.27	7.30	0.42
	Standard risk	Ref			
Radiation?	Yes	-6.14	-22.45	10.17	0.45
	No	Ref			
BMT?	Yes	-16.39	-37.93	5.16	0.13
	No	Ref			
Undergoing Treatment?	Yes	2.09	-5.70	9.87	0.60
	No	Ref			
Accumulated Present Days	Units=1 day	-0.05	-0.16	0.06	0.34
Cytarabine	Units=100 mg/m^2	0.04	-0.06	0.14	0.41
Asparaginase	Units=100[b]	0.00	-0.03	0.03	0.98
Steroids	Units=10[c]	0.04	-0.06	0.13	0.44
Anthracyclines	Units=10[d]	0.32	-0.62	1.25	0.50
Cumulative Cytarabine IT	Units=10 mg	0.51	-0.30	1.31	0.21
Cumulative Methotrexate IV	Units=100 mg/m^2	0.03	-0.02	0.08	0.29
Cumulative Methotrexate IT	Units=10 mg	0.23	-0.15	0.62	0.23
Cumulative Vincristine IV	Units=10 mg/m^2	1.34	-0.37	3.06	0.12
Age at treatment initiation	Units=1 year	-0.27	-2.27	1.72	0.78
Age at end of treatment	Units=1 year	-0.11	-2.07	1.85	0.91

[a]Adjusted for grade;

[b]100 IU/m^2 for Asparaginase IM, L-Asparaginase IM, PEG-Asparaginase; 6000 IU/m2 for Erwinia;

[c]10 mg/m^2 Dexamethasone PO; 62.5 mg/m2 Prednisone PO; [d]10 mg/m2 Doxorubicin IV; 20 mg/m^2 Daunorubicin IV.

SUPPLEMENTAL TABLE 5 Univariate analyses for science scores.

Covariate[a]	Level	Beta	95% CI		*P*
Free/Reduced Lunch?	Yes	-7.79	-16.78	1.19	0.09
	No	Ref			
Sex	Girls	-8.03	-21.85	5.79	0.25
	Boys	Ref			
Risk Stratification	High risk	-3.29	-17.36	10.79	0.64
	Standard risk	Ref			
Radiation?	Yes	3.76	-14.45	21.97	0.68
	No	Ref			
BMT?	Yes	-13.39	-37.39	10.62	0.27
	No	Ref			
Undergoing Treatment?	Yes	6.51	-3.70	16.73	0.21
	No	Ref			
Accumulated Present Days	Units=1 day	-0.04	-0.17	0.09	0.54
Cytarabine	Units=100 mg/m^2	0.10	-0.01	0.20	0.08
Asparaginase	Units=100[b]	-0.02	-0.06	0.01	0.22
Steroids	Units=10[c]	-0.01	-0.12	0.11	0.92
Anthracyclines	Units=10[d]	-0.12	-1.32	1.08	0.85
Cumulative Cytarabine IT	Units=10 mg	0.76	-0.14	1.66	0.10
Cumulative Methotrexate IV	Units=100 mg/m^2	0.04	-0.02	0.11	0.20
Cumulative Methotrexate IT	Units=10 mg	0.08	-0.43	0.58	0.76
Cumulative Vincristine IV	Units=10 mg/m^2	0.73	-1.49	2.95	0.52
Age at treatment initiation	Units=1 year	1.02	-1.20	3.24	0.36
Age at end of treatment	Units=1 year	1.33	-0.84	3.50	0.23

[a]Adjusted for grade;

[b]100 IU/m^2 for Asparaginase IM, L-Asparaginase IM, PEG-Asparaginase; 6000 IU/m2 for Erwinia;

[c]10 mg/m^2 Dexamethasone PO; 62.5 mg/m2 Prednisone PO; [d]10 mg/m2 Doxorubicin IV; 20 mg/m^2 Daunorubicin IV.
